# A Novel Predicted Calcium-Regulated Kinase Family Implicated in Neurological Disorders

**DOI:** 10.1371/journal.pone.0066427

**Published:** 2013-06-28

**Authors:** Małgorzata Dudkiewicz, Anna Lenart, Krzysztof Pawłowski

**Affiliations:** 1 Warsaw University of Life Sciences, Warsaw, Poland; 2 Nencki Institute of Experimental Biology, Polish Academy of Sciences, Warsaw, Poland; Children's Medical Research Institute, Australia

## Abstract

The catalogues of protein kinases, the essential effectors of cellular signaling, have been charted in Metazoan genomes for a decade now. Yet, surprisingly, using bioinformatics tools, we predicted protein kinase structure for proteins coded by five related human genes and their Metazoan homologues, the FAM69 family. Analysis of three-dimensional structure models and conservation of the classic catalytic motifs of protein kinases present in four out of five human FAM69 proteins suggests they might have retained catalytic phosphotransferase activity. An EF-hand Ca^2+^-binding domain in FAM69A and FAM69B proteins, inserted within the structure of the kinase domain, suggests they may function as Ca^2+^-dependent kinases. The FAM69 genes, *FAM69A, FAM69B, FAM69C, C3ORF58 (DIA1)* and *CXORF36 (DIA1R),* are by large uncharacterised molecularly, yet linked to several neurological disorders in genetics studies. The *C3ORF58* gene is found deleted in autism, and resides in the Golgi. Unusually high cysteine content and presence of signal peptides in some of the family members suggest that FAM69 proteins may be involved in phosphorylation of proteins in the secretory pathway and/or of extracellular proteins.

## Introduction

In pre-genomics biology, defined molecular function in search of a protein effector was a common reality. The opposite, a protein in search of a function is still an even more common problem in biology.

Protein kinase-like (PKL) proteins are a huge grouping of regulatory/signalling and biosynthetic enzymes [1], [2], regulating most processes in a living cell, by phosphorylating various substrates. Besides PKL kinases, other kinase families are known, functionally related, but of dissimilar structures [3]. Most PKL proteins feature a well-conserved structural scaffold, and a conserved active site [4], [5]. These classic protein kinases number more than 500 in the human genome [1], and are among the most popular drug targets [6]. Within the protein kinase-like clan, sequence similarities between some families are relatively low. There are also PKL-like families that may be assigned to the clan mostly by the virtue of structural similarity. Despite the high interest in kinases (and overall almost six hundred thousand PubMed articles as of September 2012), the research effort has been biased [7], [8] whereas approx. 10% of known kinases yielded at least 90% of publications [9]. Also, the human, and more generally, Metazoan kinome may be not fully charted yet. For example, a protein kinase-like domain has been recently discovered in selenoprotein O [10]. Also, a novel protein kinase family, FAM20, has been identified and characterised, [11]–[14], involved in phosphorylation of secreted proteins.

High-throughput studies often lead to discovery of disease links for uncharacterised proteins that due to lack of molecular function hypotheses are not followed upon. Here, using bioinformatics approaches, we analyse an obscure group of human proteins with disease implications. First, we prove that FAM69 family members are homologous to protein kinases. Second, we predict that most FAM69s do have protein kinase activity. Third, we predict that some FAM69s are directly regulated by calcium ions via an EF-hand domain inserted in the middle of the kinase domain and close to the ATP-binding site. Fourth, we hypothesize that FAM69s may in fact regulate secretory pathways.

## Results

### FAM69s Belong to the Protein Kinase-like Clan

Examination of protein families with sequences distantly similar to the classical protein kinases, according to results of the FFAS algorithm [15], suggested that the uncharacterised Pfam family PIP49_C (Pfam:PF12260, Pancreatitis induced protein 49 C terminal) [16] might be homologous to protein kinases. Indeed, using a member of the PIP49_C family, the human FAM69A protein as a query, in the second PSI-Blast iteration [17] one detects significant sequence similarity to a human Ser/Thr protein kinase PKDCC (gi:292495024), with a significant E-value parameter equal to 3E-07, although with only 21% sequence identity over 197 residues. The distant albeit significant sequence similarity to both Ser/Thr kinases and to Tyr kinases does not allow for an unequivocal distinction between these two possibilities. Also, FFAS and HHpred structure prediction algorithms provide highly significant kinase-like predictions: Zscore -20 and E-value 1E-26, respectively. Among the structural predictions, Ser/Thr kinases are most common. The significant sequence similarity covers approximately the region 225–415 of the human FAM69A protein, that aligns to the C-terminal lobe of the kinase domain and the β-4 β-5 region of the N-terminal lobe.

Since no equivalent of the ATP-binding glycine-rich loop which is typical for kinases was found in FAM69s, the N-terminal region of FAM69 was analysed separately. The region between residues 75 and 130 in human FAM69A exhibited sequence similarity of borderline significance to N-terminal lobe of protein kinases (FFAS Zscore −7.6 and HHpred P-value 1E-05). Secondary prediction for this region supported the 3D structure prediction.

**Figure 1:**
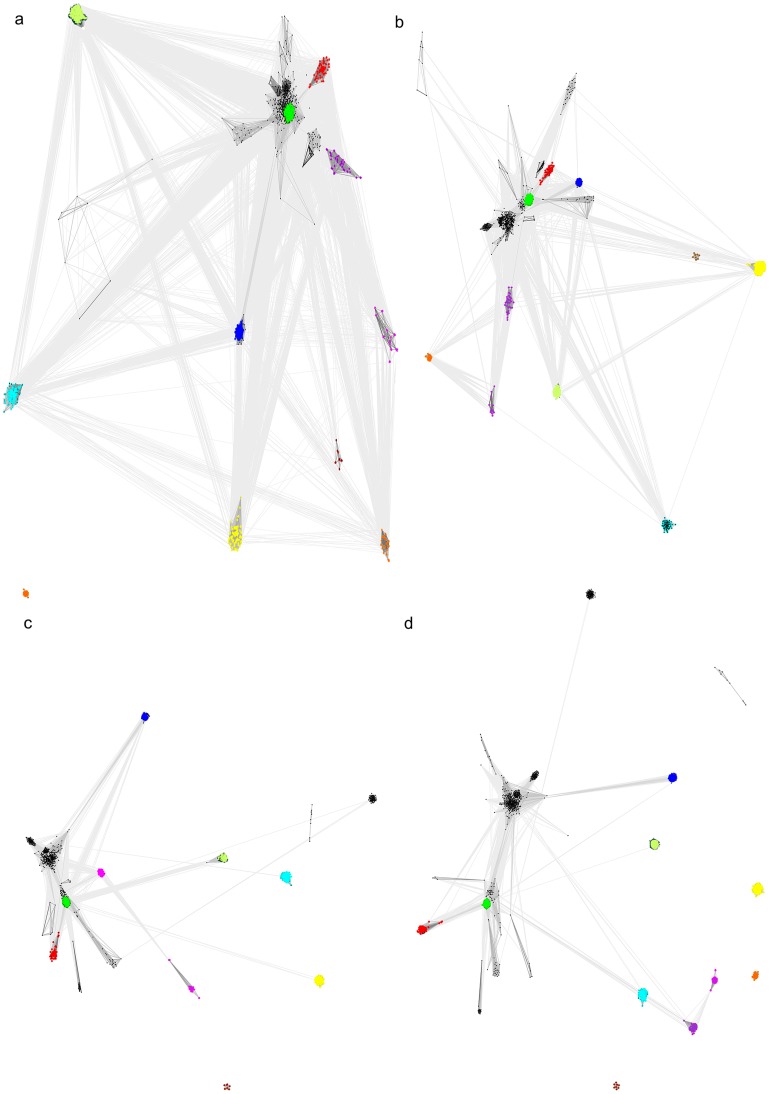
CLANS graphs visualizing sequence similarities between protein kinase-like families. Nodes represent sequences, edges represent similarity relationships. PSI-BLAST-detected significant (dark grey) and sub-significant (light grey) similarities shown. Dark green: pkinase and pkinase_Tyr families, Red: FAM69; dark blue: SELO; yellow: alpha kinase; brown: UL97; cyan: PPDK, magenta: PI3_PI4; light green: PIP5K, orange: DUF1193 (FAM20); Black: other kinase families. The following P-value thresholds for significance of sequence similarity were used: 0.1 (*top left*), 0.001 (*top right*), 1E-5 (*bottom left*) and 1E-10 (*bottom right*).

CLANS clustering analysis [18], presenting in a graph form distant sequence similarity relationships between groups of proteins, reflects relatively close similarity of FAM69s to many known kinase families (Fig. 1). Specifically, regardless of the sequence similarity clustering threshold used, FAM69s remain grouped with the central group of kinase families, including the classic pkinase and pkinase_Tyr (Pfam database identifiers: PF00069, PF07714) families. Of note, some of the kinase families, members of the PKL clan for which homology to typical PKL proteins has been established, e.g. UL97 (PF06734), are found to be more distant from the central families than FAM69s (see Fig. 1).

**Figure 2:**
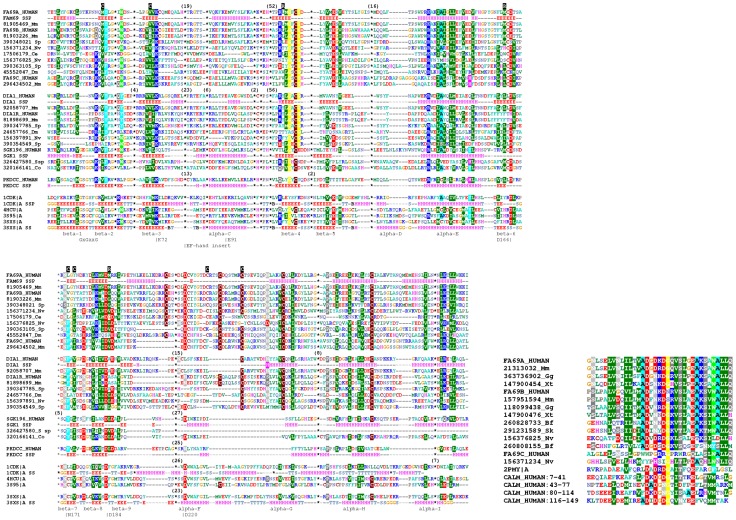
Multiple sequence alignments of selected FAM69 proteins. *Left:* alignment of the kinase domain, covering the region 83–422 of FAM69A. The sequences are aligned using Promals3D (see Methods). Secondary structure prediction for human shown for selected proteins, for the solved structures actual secondary structure shown. Secondary structure elements named as in PKA [87]. Locations of predicted key catalytic residues shown, in standard PKA numbering (e.g. D166), as well as the ATP-binding loop (GxGxxG). SwissProt identifiers shown for human sequences, otherwise, NCBI GI identifiers shown together with abbreviations of species names: Sp: sea urchin *Strongylocentrotus purpuratus, Nv:* sea cucomber *Nematostella vectens, Dm:* fruit fly *Drosophila melanogaster; Mm: Mus musculus; Ce: Caenorhabditis elegans, Co: Capsaspora owczarzaki; S sp: Salpingoeca sp. ATCC 50818*. Also shown selected close kinase homologues (PKDCC and SGK196) as well as sequences of selected kinases of known structures. Numbers in brackets indicate numbers of residues omitted from the alignment (shown only for the 1 cdk, 3 sxs structures, and for human FAM69A, DIA1, PKDCC and SGK196 sequences). R and C characters on black background above the alignment indicate the regulatory and catalytic spine residues, respectively [22]. The location of the the EF-hand motif shown, the motif itself is excised from the alignment and shown on the right. *Right:* alignment of the EF-hand region (corresponding to the region 165–199 of human FAM69A). Also shown EF-hand regions of human calmodulin and the 2PMY region used for model building.

The FAM69 family is present in most Metazoan taxa, with the sea anemone *Nematostella vectensis* being the organism most distant from humans possessing it. *N. vectensis* has four FAM69 genes, three of which code proteins with the typical FAM69 features conserved (See Fig. 2, left). FAM69s can be clearly separated into two major branches, one containing human FAM69A, FAM69B, FAM69C and the other containing C3ORF58 (DIA1) and CXORF36 (DIA1R) (See Fig. S1). The two protein groups have been analysed bioinformatically [19], [20], but they have never been identified as homologues before, although their sequence similarity to each other is very significant (FFAS Zscore equals −56 and HHalign E-value equals 3E-12 between human FAM69A and C3ORF58, see also Fig. 2, left).

### Most FAM69s are Predicted to be Active Kinases

Structure predictions are not automatically extendable to function predictions. Here, we will look more closely at the sequence and structure motifs important for kinase function.

FAM69s exhibit strong and significant sequence similarity to the C-terminal lobe of the classical kinase structure. Sequence similarity of FAM69s to the N-terminal smaller lobe of kinases is of borderline significance. Yet, after removal of the insertion sequence forming the EF-hand motif (approx. residues 165–190, see the following section), the remaining regions of FAM69A exhibit significant overall similarity to protein kinases (HHpred E-values below 1E-33).

**Figure 3:**
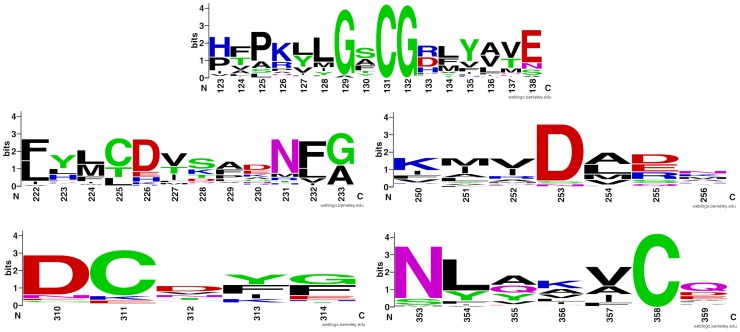
Kinase motifs. Sequence logos for selected kinase motifs in the FAM69 family. *Top:* the β-4 - β-5 region. *Middle row, left:* the predicted active site region (corresponding to D166 and N171 in PKA). *Middle row, right*- the Mg^2+^ - binding motif (corresponding to the DFG motif in PKA). *Bottom, left:* predicted helix α-F. *Bottom, right:* predicted helix α-G.

Several of the conserved kinase regions, as defined for the archetypical protein kinase A (PKA) [21], are clearly conserved in FAM69s (See Fig. 2, left). The presumed catalytic base (D166 in PKA) is present in most FAM69s, excluding CXORF36, albeit in a [LM][CL]D motif (D294 in human FAM69A) instead of [HY]RD. Also well-conserved are residues corresponding to N171 and D184 of PKA, responsible for binding the Mg^2+^ ions (N299 and D312 in human FAM69A). Then, conserved are residues corresponding to K72 and E91 in strand β-3 and helix α-C, respectively (K113 and E151 in human FAM69A) involved in binding the phosphate groups of the ATP molecule (see sequence logos in Fig. 3). However, the identification of the location of helix α-C is less reliable than the identification of β strands 1, 2 and 3. As another possibility, the helical region following the predicted helix α-C (see next section) may in fact provide an alternative location for a glutamate residue corresponding to the E91 of PKA kinase.

In cases of remote sequence similarity, building a structural model is of an illustrative nature rather than predictive, yet it serves also as a feasibility check for the predicted structure. We analysed a structural model of the FAM69 kinase domain (residues 77–423, including the EF-hand domain region) built using selected kinase structures as templates (see Methods) and a manually curated sequence alignment (See Fig. S2). Overall, the structure model is reasonable as judged by the MetaMQAP model quality scoring (GDT_TS parameter equal to 42.7 and expected Cα RMSD from native structure is 4.8 Å).

The core structure of the protein kinases contains an unique ATP-binding motif, GxGxxG. In FAM69s, structure prediction algorithms do not allow unambiguous detection of a corresponding site. However, an analysis of weak fold predictions (FFAS and HHpred, see Methods), together with secondary structure predictions allowed detection of similarity to the kinase N-terminal lobe. Since the typical kinase Gly-rich motif is not present in FAM69s in the predicted β-1–β-2 loop, it is likely that an atypical ATP-binding mode is employed in the FAM69 family, possibly using the partly conserved proline residue present in that loop in FAM69A and FAM69B, or the glycine residue that replaces it in FAM69C homologues.

The recently identified kinase features, the regulatory and catalytic spines built of conserved hydrophobic residues [22], are not easily identified in full in FAM69s due to substantial sequence divergence. Yet, most of these residues can be tentatively identified. In the regulatory spine, only L95 (PKA numbering) of helix α-C cannot be easily identified in FAM69s. Otherwise, regulatory spine residues are conserved (see Fig. 2, left), including L106 of strand β -4, Y164 next to the predicted active site Asp, and F185 next to the Mg^2+^-binding Asp184. Among the catalytic spine residues, V57 of strand β-2 is conserved, as well as A70 of strand β-3 and L172 and I174 located next to the predicted active site. The neighbouring L173 is usually not conserved as a hydrophobic residue. Finally, the M228 and M231 residues of helix α-F are a difficult case due to the relatively uncertain sequence alignment for this region. However, interestingly, provided our alignment is realistic, these two residues are replaced by cysteines in FAM69A, B and C homologues which opens up a possibility that a disulphide bridge between these two residues stabilises the catalytic spine (See Fig. 2, left). Structural predictions for uncharacterised proteins are an important approach for advancing molecular biology and suggesting specific expermental approaches guided by the predictions [23]–[25]. Such approaches have been repeatedly successful. For example, the CLCA family was predicted to possess peptidase function, and later confirmed experimentally [26], [27]. Likewise, the NLRP proteins were predicted to be involved in protein-protein interactions in immune responses and apoptosis, which has been subsequently validated [28], [29]. The usefulness of a structural prediction depends on both the accuracy and the purpose of the prediction. It has been shown that inclusion of explicit water molecules greatly improves the quality of structural models and information derived therefrom [30], [31]. However, in remote homology-based predictions like this one, that aim at general function prediction and suggesting functional validation experiments, the purpose of actual structural model building is illustrative rather than predictive. Hence, water is not explicitly included.

The FAM69 proteins contain many cysteine residues (between 12 and 18 in human proteins), most of them conserved (see Fig. 2, left) and it has been suggested that most of them participate in disulphide bridges [20]. In our structure model of human FAM69A, some plausible S-S bridges can be postulated. One involves Cys293, next to the predicted active site aspartate D294, bridged to Cys331 (HCE motif located between strand β-9 and helix α-F, in the region corresponding to the kinase activation loop) and possibly stabilising the predicted active site conformation and positioning of D294. After manual adjustment of sidechain torsions of the two cysteine residues in the model, the sulphur-sulphur distance is 4.8 Å. Other possible disulphide bridges may involve six cysteine residues located within or near helices α-F, α-G and α-H and could stabilise the C-terminal lobe of FAM69s. In the FAM69A structure model, the sulphur atoms of Cys243 and Cys293 (from the DCR and SCI motives, located in helices α-F and α-H, respectively) are located within 3.19 Å. Further probable disulphide bridges could stabilise the N-terminal lobe and the ATP-binding region. Of note, some of cysteine residues conserved in the FAM69 family are also found in PKDCC and SGK196 kinases (see Fig. 2). The cysteine residue of helix α-G is also conserved in many other known kinases, including the 4hcuA, 3sxsA and 3s95A structures (see Fig. 2, left).

Nevertheless, the cysteine-rich kinase domain of FAM69s seems to be a unique arrangement among the known kinases. In the 1354 representatives of 27 kinase and putative kinase families as shown in Fig. 1, family-wide averages of cysteine residue count per kinase domain ranged from 0.6 to 10.6. Standard Analysis of Variation (ANOVA) test shows that the FAM69 family stands out as significantly different from most other kinase families when kinase domains are compared for cysteine content (ANOVA p-value is less than 10^−4^). FAM69 forms a homogenous group together with the viral UL97 family [32], [33]. These viral kinases function intracellularly, in the nucleus and in the cytoplasm, facilitating viral infection by interfering with cellular processes such as cell cycle regulation and DNA replication. Of interest, the UL97 kinases have unusual optimal conditions for their catalytic activity (1.5 M NaCl and pH 9.5) [33]. Their high cysteine content may be related to these atypical preferred catalytic conditions. A kinase family following next, when sorted by cysteine count, is the DUF1193 (FAM20) [13], [14]. This agrees with both FAM20 and FAM69 being known or predicted, respectively, to be extracellular. It has been accepted for a long time that, in general, extracellular proteins have higher cysteine and cystine content than intracellular ones [34]–[36].

**Figure 4:**
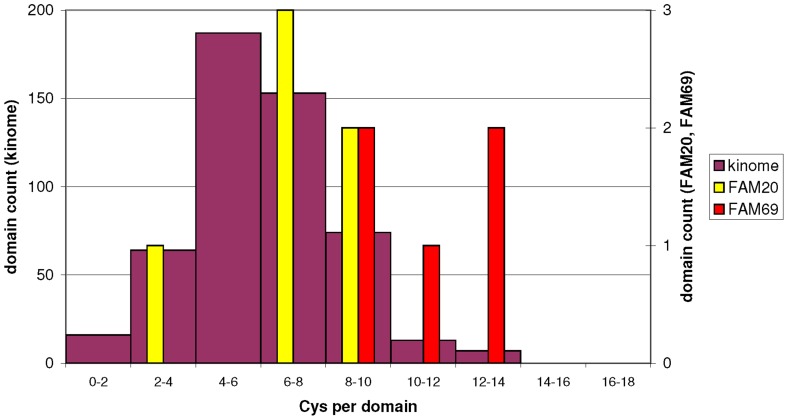
Cysteines in kinases. Histogram of cysteine residue count in kinase domains. Left scale, magenta bars: 516 kinase domains of the human kinome [1], Right scale, red bars: human FAM69 kinase domains; right scale, yellow bars: human FAM20 kinase domains.

When the human kinome alone [1] is analysed for cysteine content, again the FAM69 family stands out. In the chart (see Fig. 4), the FAM69 predicted kinase domains are found at the far right tail of cysteine count distribution. The difference between FAM69 and the established kinome is even more pronounced, because cysteine counts in kinase domains are imprecise due to imprecise definitions of domain boundaries. For example, some of the kinase domains with most cysteine counted (e.g. CDC7 and SRPK1) in fact possess Cys-rich insert regions within the kinase domain.

In contrast to FAM69, some of the known kinases that are termed cysteine-rich [37]–[39] are actually ones that contain specific cysteine-rich domains next to their kinase domains. In contrast, in the FAM69 family, the numerous cysteine residues are located within the kinase domain itself.

### Some FAM69s Contain a Ca^2+^-Binding Motif

**Figure 5:**
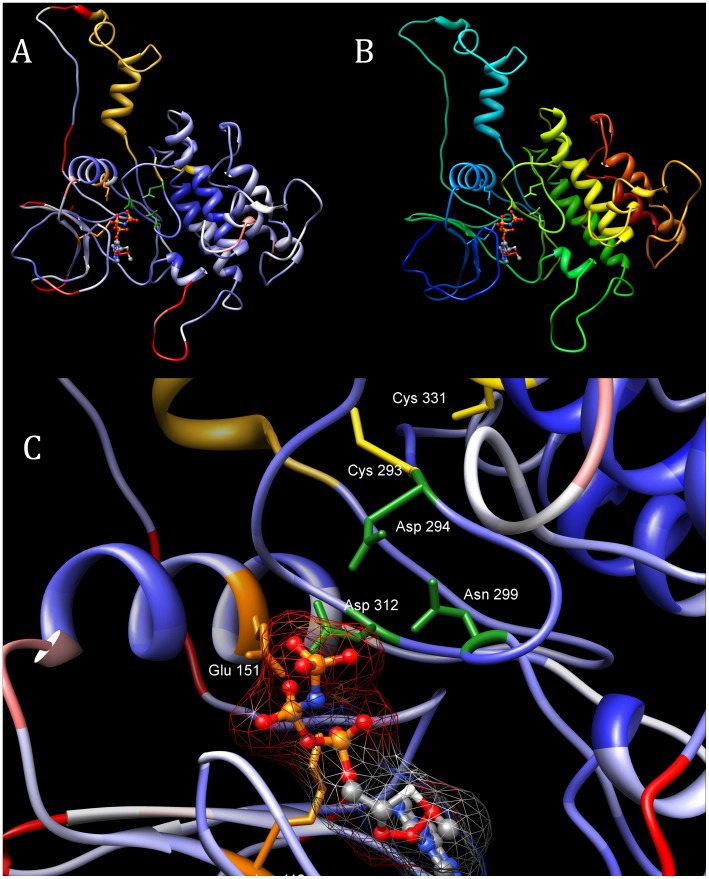
Structure model of the kinase domain of human FAM69A. *Top left:* model coloured by MetaMQAP model quality score (blue: good quality, red: poor quality). On left, the EF-hand motif is shown in yellow. *Top right:* as in Fig. 5 (top left), model coloured by sequence: from dark blue (N-terminus) to dark red (C-terminus). *Bottom:* close-up of the predicted active site with ATP molecule bound. Side chains of key predicted active site residues shown: D294 (PKA numbering: 166), N299 (171), D312 (184), also the two cysteines near the predicted active site that may form a S-S bridge: C293 and C331.

Close to the predicted kinase ATP-binding loop in human FAM69A, a single EF-hand Ca^2+^-binding motif can be detected by the HHpred algorithm, with p-value 1E-11, in the region 165–190, approximately. The motif, inserted within the N-terminal lobe of the kinase domain, most likely between helix C and strand β*-*4, is easily detected only in some FAM69s, e.g. human FAM69A and FAM69B, as well as one *Nematostella vectensis* protein, gi: 156376825, see Fig. 2 (right). In these proteins, the motif features all the residues necessary for the Ca^2+^-binding activity, i.e. DxDxDGx[IV]xxxE in the ion-binding loop [40]. In other FAM69 sequences, including the other human sequences (FAM69C, C3ORF58 and CXORF36), some features of the EF-hand are visible, albeit due to many substitutions they are unlikely to bind calcium ions (See Fig. 2, right). To our knowledge, FAM69 is the first case of a protein-kinase like domain fused with an EF-hand motif in *Metazoa.* However, similar domain combinations are known in plants and some protists [41]–[43]. Yet, there, the EF-hands are located next to the kinase domains, not inserted into them. In humans, many protein kinases are regulated by calcium, but either by interaction with independent calcium sensors, e.g. calmodulin or by utilising specialised calcium-binding domains, C2, unrelated to EF-hands [44]. The unique location of the EF-hand domain within the FAM69 kinase domain, between helix α-C and strand β-4 and near the predicted ATP site and predicted active site (see Fig. 5), suggests a regulatory role. This is supported by very good evolutionary conservation of the EF-hand domain in members of the FAM69 family from vertebrates and distant Metazoa, e.g. *Nematostella*. Since EF-hand motifs undergo Ca^2+^-dependent dimerisation, one may speculate that FAM69A and FAM69B kinase activity depends on Ca^2+^-binding-induced dimerisation.

### FAM69s are Implicated in Neurological Disorders

According to the PolyPhobius algorithm, FAM69A, B and C proteins possess transmembrane regions between residues 20–50, while C3ORF58 and CXORF36 have signal peptides (See Fig. S3). Very similar predictions are obtained using the TMHMM tool. The TMHMM algorithm predicts extracellular location for all the five human FAM69 proteins (except the short cytoplasmic N-termini of FAM69A, B and C). The FAM69A, B and C proteins have been reported to localise to endoplasmic reticulum (ER) [20] as putative membrane-anchored molecules, while C3ORF58 (DIA1, GoPro49) has been shown to reside in the Golgi [45]. Tissue-wise, expression of FAM69A is ubiquitous while FAM69B and FAM69C are expressed mostly in the brain, the latter also in the eye [20].

C3ORF58 expression was observed in cartilaginous mesenchymal tissues, regulated developmentally, with highest expression seen in proliferating chondrocytes [45]. Further, colocalization with beta-coatomer protein was seen, suggestive of a function in membrane traffic [46]. Then, characteristic expression of C3ORF58 was observed in dental follicles, again suggestive of a role in trafficking and secretion [46]. The *CXORF36* gene has an ubiquitous expression pattern. The expression patterns of FAM69 genes and proteins, including brain, dental follicles, developing mesenchyma and cartilaginous cells, could be reconciled if one assumed participation in biological processes where substantial secretory activity is essential.

Consistently with brain-specific or brain-including expression pattern, several FAM69 genes were implicated in a number of neural disorders. One of two largest chromosome region deletions in autism involves the *C3ORF58 (DIA1)* gene [47]. Of note, *C3ORF58* is up-regulated by neuronal activity, as shown by MEF2 RNAi assay [47].

The *CXORF36* (*DIA1R)* gene has been linked to the fragile X syndrome (FXS), with non-synonymous mutations found in this gene in two studies: S24P, K128R [48], [49]. The molecular mechanisms underlying FXS are overlapping with those responsible for autism, since 30% of FXS patients develop autism [50]. In several publications, the Xp11.3 region that includes *CXORF36* has been linked to neurological disorders [51], including X-linked mental retardation (XLMR). Further, a gene in Xp11.3–4 region may contribute to the higher autism susceptibility in men [52]. Finally, recently, deletion of *CXORF36* was observed in the Kabuki syndrome, a congenital mental retardation syndrome [53].

The *FAM69A* gene has been linked to schizophrenia and bipolar disorder, with two intronic significant SNPs identified in a meta-analysis [54]. Also, the *FAM69A* region is the risk locus for multiple sclerosis, although other genes in that region may be the primary culprits [55].

An analysis of rare copy number variation (CNV) in autism spectrum disorders found variation in three FAM69 genes: *FAM69B, C3ORF58, CXORF36* -2028680287 [56]. *A*lso, a deletion of *FAM69B* in autism has been observed [57]. A network-based analysis of genes with CNV in autism identified involvement of synapse formation and function processes [58].

## Discussion

We present the discovery of a novel putative kinase family with members in humans and presence throughout *Metazoa* as a yet another small step towards filling in the blank spots in the complex regulatory machinery of the living cell. Charting of the kinome is important for unbiased advancement of biology and medicine [9], [59].

Can the kinase function prediction for FAM69s be trusted, or is it only a reliable three-dimensional fold prediction? Conservation of key residues and evolutionary conservation in Metazoa suggest indeed a conserved kinase function. For very distant homologues, the sequence alignment details are known to be less reliable than the overall detection of homology [60]. Thus, some of our definitions of FAM69 predicted active site motifs (e.g. location of the residue corresponding to E91 of PKA) or secondary structure element assignments may be erroneous. Also, it is not straightforward to predict the substrate, yet, FAM69 similarity to classic protein kinases suggests FAM69 proteins are kinases that phosphorylate proteins. An exception is one of the FAM69 proteins, CXORF36 (DIA1R), a protein restricted to vertebrates (see Fig. S1) [61]. Although it clearly is a homologue of C3ORF58 (DIA1), it does not have the predicted active site aspartate conserved (corresponding to D166 in PKA). Thus, CXORF36 is probably a pseudokinase that may interfere with signalling by other FAM69 proteins in a dominant negative fashion. Alternatively, CXORF36 may be a highly atypical kinase.

Precise placement of FAM69s in the protein kinase clan is difficult due to the presence of the EF-hand insert and sequence divergence (see Fig. 1). Closest known kinase homologues seem to be the PKDCC and the SGK196 subfamilies of the pkinase and pkinase_Tyr families (see Fig. S4). The uncharacterised SGK196 kinase group is present in Opisthokonts (*Metazoa*, *Choanoflagellates*, and the early-branching opisthokont *Capsaspora,* but not *Fungi*). It also has remote homologues in plants. Of note, the atypical MCD motif found in the predicted active site of FAM69A instead of [HY]RD (the catalytic motif of typical kinases), is present also in most SGK196 homologues, including a primitive unicellular opisthokont, filasterean *Capsaspora owczarzaki* [62], [63], a choanoflagellate, *Salpingoeca* sp. ATCC 50818 [64], and one of simplest multicellular animals, the plocozoan *Trichoplax*. Similarly to FAM69A, B and C, the SGK196 protein is predicted to be an extracellular protein possessing an N-terminal transmembrane segment. The second protein group clearly homologous to FAM69, is the PKDCC subfamily (protein kinase domain containing, cytoplasmic; SgK493; Vertebrate lonesome kinase, VLK [65]). Contrary to the VLK designation (vertebrate-specific), PKDCC is present in Metazoa (e.g. *Nematostella, Strongylocentrotus*). PKDCC is enriched in the Golgi apparatus and regulates transport from the Golgi to the plasma membrane [66]. On a systemic level, PKDCC regulates lung and bone development [65], [66]. Lack of PKDCC results in morphological abnormalities, such as linked to deficient biomineralisation [65]. Recently, other Golgi-localised kinases have gained interest, namely the recently discovered Metazoan FAM20 kinases (FAM20C, FAM20B and Four-Jointed) that are not closely related to FAM69s by sequence similarity. These kinases have been demonstrated to phosphorylate secreted proteins (or xylose in the case of FAM20B). FAM20B and Four-Jointed reside in the Golgi while FAM20C has been observed both in the Golgi and extracellularly.

Taking together the information on FAM69 predicted kinases, it is all too tempting to speculate that indeed, FAM69s carry out functions similar to the PKDCC and FAM20 novel kinases [11]–[14], [66]. Thus, different FAM69 proteins may be involved in phosphorylation of secreted proteins, or in regulating the transport from ER to Golgi and from Golgi to the plasma membrane. Thus, one may speculate that some of FAM69s may embody the yet unidentified kinases regulating of ER-to-Golgi vesicle transport [67,2011,H89]. It is recognised that vesicular trafficking is critical in neuron development and its malfunctions may result in mental retardation [68]. Thus, the neurological disorders related to FAM69 genes may have a common denominator, malfunction of the secretory pathway in neurons [19].

Evolutionary origin of FAM69 predicted kinases is obviously within the protein kinase-like (PKL) clan. In contrast to FAM69s, PKDCC and SGK196 clearly belong to the classic pkinase and pkinase_Tyr families, respectively (Pfam database identifiers PF00069, PF07714), according to the HMMER tool (see Fig. S4). Because the phylogenetic spread of the SGK196 subfamily is broader than the spread of PKDCC subfamily of the FAM69s, one may speculate that the FAM69 family originated from a SGK196-like ancestor early in Metazoan evolution. It has been noted previously that kinase repertoire essential for multicellular life originated in pre-Metazoan unicellular Eukaryotes [69].

Translating a structure prediction into a useful function prediction is a challenge. Here, we strove to achieve this, complementing structure predictions with analyses of available functional data and literature. Yet, the ultimate answers as to the functions of the FAM69 family will only come from experiments.

## Methods

For remote homology identification, PSI-BLAST searches used the standard parameters on nr database at NCBI as of 09.2012. For domain assignments, HMMER3 [70] on the Pfam database as of 09.2012 was used.

For survey of similarities within the kinase-like clan (Fig. 1), the CLANS algorithm [18] was run on a set of sequences including a) all Pfam seeds from the 17 families of the protein kinase-like clan (CL0016), b) the seeds from FAM69 family (PIP49_C, Pfam:PF12260), c) representative SELO domains [10], d) seeds from the Pfam families: Alpha_kinase (PF02816), PI3_PI4_kinase (PF00454), Act-Frag_cataly kinase, PF09192, PPDK_N (PF01326), PIP5K (PF01504), e) the DUF1194 family (FAM20) [12], [14]. For the d) group, structural similarity to the PKL kinases is known [3]. CLANS was run with 5 iterations of PSI-BLAST, using the BLOSUM45 substitution matrix and inclusion threshold 0.001. For the CLANS graphs, sequence similarity relations with significance of P-values below 0.1, 0.001, 1E-5 and 1E-10 were considered, as indicated in the Figure 1.

For closer examination of similarities between FAM69 and other kinases (Fig. S4), a set of representative sequences was built as follows. The kinase domain regions of the human FAM69A, DIA1, PKDCC and SGK196 proteins (as outlined in Fig. 2, left) were used to find representative homologous sequences by running one iteration of JackHMMER [71] on the RefSeqP database with 1E-5 E-value threshold. The representative sequences found were cleared of redundancy above 70% sequence identity using CD-HIT [72]. Then, the sequences obtained plus seeds for the pkinase and pkinase_Tyr Pfam families were input to the CLANS algorithm, run on nr90 and env_nr90 databases with the Blosum45 matrix and 3 iterations of PSI-Blast. The cutoff for inclusion of a relation in the CLANS graph building was P-value equal to 1E-5.

Transmembrane region predictions were achieved by the TMHMM and Phobius servers [73], [74]. The Jpred and PsiPred servers were used to predict the secondary structures [75], [76]. Multiple sequence alignments of FAM69A, B, C and DIA1, DIA1R subfamilies were built using the MUSCLE program [77]. The final multiple sequence alignment was built using Promals3D, with additional alignment constraints for selected kinase motifs [78]. The constraints were derived from HHpred alignments of human FAM69A, DIA1, PKDCC and SGK196 to the 3sxsA sequence. HHpred was run using MUSCLE-generated alignments of close homologues of the above-listed four proteins.

The structure of human FAM69A was modelled by comparative modelling. The templates were chosen based on results of FFAS03 predictions run on PDB database. The FFAS03 method [15] that uses sequence profile-to-profile comparison was supplemented by the HHpred algorithm [79] that employs HMM-to-HMM comparison. Best scoring kinase structures (FFAS03 score below −20 and percent identity above 9) were picked up for further analysis. The modelled (target) structure was constructed based on pairwise alignments of FAM69A sequence and the two templates identified by FFAS03 (3HGK_A: kinase Pto from *Solanum pimpinellifolium* [80] and 3S95_A: human LIMK1 kinase domain [81]). Since analysis of FAM69A sequence indicated the presence of the EF-hand Ca^2+^-binding motif, which is absent in typical kinases, the model was constructed using combined templates, incorporating an EF-hand domain into the template. According to FFAS03 score, the 2PMY structure (EF-domain of human RASEF, region 59–89) was selected for modelling purposes. Because the selected templates do not contain ATP ligands, the structure of protein kinase A complexed with ANP and Mn^2+^ (PDB:1CDK) was used to identify the possible ANP and Mn^2+^ ligand positions in 3HGK structure and to manually place ANP structure in the model. The two kinase templates were aligned using FATCAT (Flexible structure alignment by chaining aligned fragment pairs allowing twists) [82] and the resultant alignment served for modelling. We combined FATCAT, HHpred and FFAS03 pairwise alignments to refine the multiple alignment between the modelled sequence and selected templates. The alignment was also manually adjusted to accommodate predicted secondary structures (See Fig. S2).

The structure model was constructed automatically by the program MODELLER9 v 8 [83] using the combined scripts: homology modelling with multiple templates and model-ligand.

The MetaMQAP server [84] was used to estimate the correctness of the 3D models using a number of model quality assessment methods in a meta-analysis.

For sequence logos, the WebLogo tool was used [85]. The sequences represented in the logo are obtained by the JackHMMer tool [71] using the sequence alignment from Fig. 2 (left) as query against the RefSeq database, and similarity threshold at bitscore 70 to avoid distant homologues.

For presentation of multiple sequence alignments, the BioEdit software was used [86].

## Supporting Information

Figure S1 **Phylogenetic tree of the FAM69 sequences obtained using the alignment from** **Fig. 2** **(left) and the PhyML maximum likelihood algorithm** [**88**] **. Approximate bootstrap values shown.**
(PDF)

Figure S2 **Target/template alignment used for structure modeling.**
(PDF)

Figure S3 **Transmembrane helix and signal peptide predictions for human FAM69 proteins, obtained by the Phobius algorithm.**
(PDF)

Figure S4 **CLANS analysis for FAM69, SGK196 and PKDCC proteins together with pkinase and pkinase_Tyr families.** Dark blue: FAM69ABC subfamily, light blue: DIA1 subfamily. Dark green: PKDCC homologues. Light green: SGK196 homologues. Orange: pkinase_Tyr family seeds, Red: pkinase family seeds. Sequence similarity relations with significance of P-value below 1E- considered.
(TIF)
