# Supplementary figures and images for: A Novel Predicted Calcium-Regulated Kinase Family Implicated in Neurological Disorders
Source: PLoS One. 2013 Jun 28;8(6):e66427. doi: 10.1371/journal.pone.0066427 (PMC3696010; doi:10.1371/journal.pone.0066427)

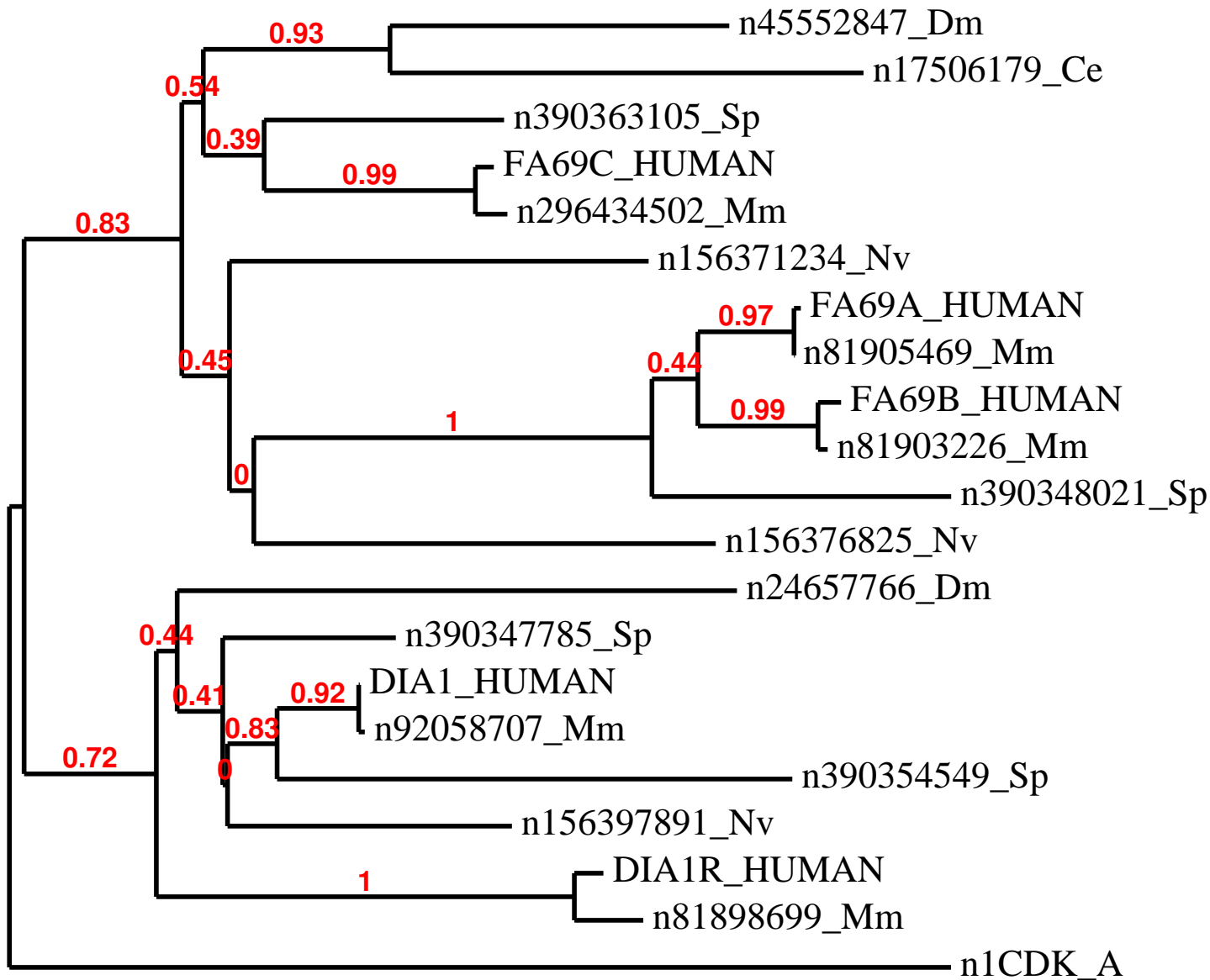

Supplement: Figure S1 — Phylogenetic tree of the FAM69 sequences obtained using the alignment from Fig. 2 (left) and the PhyML maximum likelihood algorithm [88] . Approximate bootstrap values shown. (PDF) [file pone.0066427.s001.pdf]

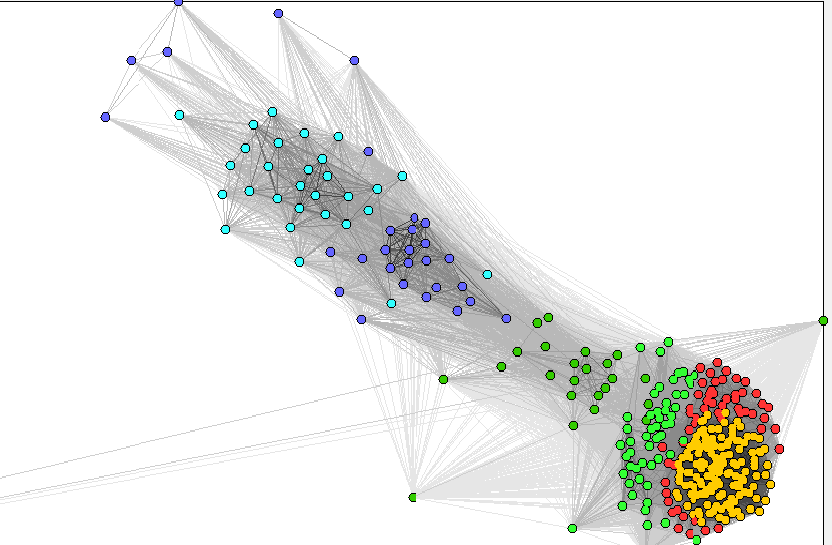

Supplement: Figure S4 — CLANS analysis for FAM69, SGK196 and PKDCC proteins together with pkinase and pkinase_Tyr families. Dark blue: FAM69ABC subfamily, light blue: DIA1 subfamily. Dark green: PKDCC homologues. Light green: SGK196 homologues. Orange: pkinase_Tyr family seeds, Red: pkinase family seeds. Sequence similarity relations with significance of P-value below 1E- considered. (TIF) [file pone.0066427.s004.tif]
