# Supplementary material for: A Novel Predicted Calcium-Regulated Kinase Family Implicated in Neurological Disorders
Source: PLoS One. 2013 Jun 28;8(6):e66427. doi: 10.1371/journal.pone.0066427 (PMC3696010; doi:10.1371/journal.pone.0066427)
Supplement: Figure S2 — Target/template alignment used for structure modeling. (PDF) [file pone.0066427.s002.pdf]

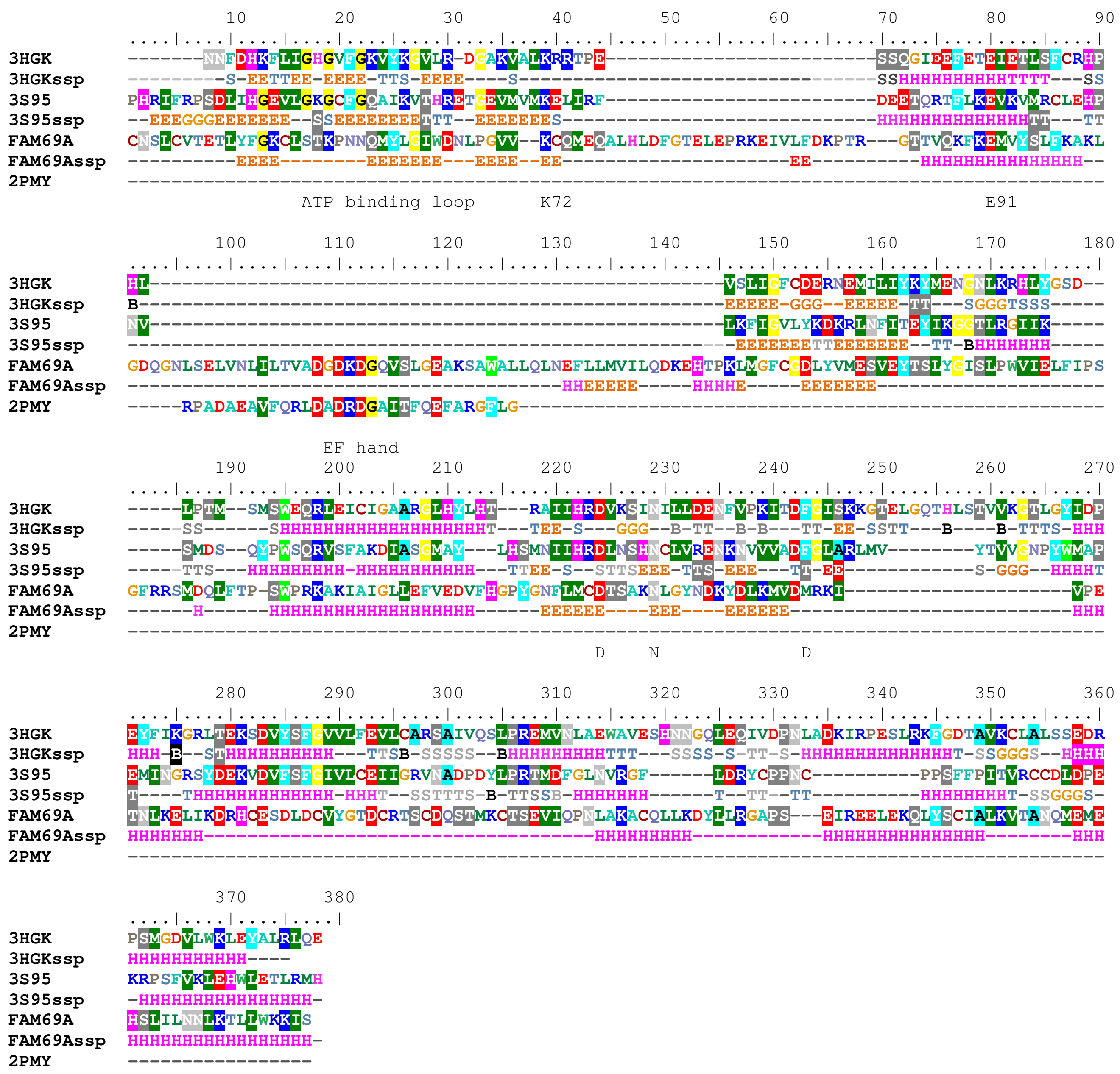
3HGK
3HGKssp
3S95
3S95ssp
FAM69A
FAM69Assp
2PMY
10 20 30 40 50 60 70 80 90
ATP binding loop
K72
E91
100 110 120 130 140 150 160 170 180
EF hand
190 200 210 220 230 240 250 260 270
D
N
D
280 290 300 310 320 330 340 350 360
370 380
