# Supplementary material for: A Novel Predicted Calcium-Regulated Kinase Family Implicated in Neurological Disorders
Source: PLoS One. 2013 Jun 28;8(6):e66427. doi: 10.1371/journal.pone.0066427 (PMC3696010; doi:10.1371/journal.pone.0066427)
Supplement: Figure S3 — Transmembrane helix and signal peptide predictions for human FAM69 proteins, obtained by the Phobius algorithm. (PDF) [file pone.0066427.s003.pdf]

PolyPhobius prediction

Prediction of gi|165932391|ref|NP_001006606.2|

```
ID   gi|165932391|ref|NP_001006606.2|
FT   TOPO_DOM      1     27       CYTOPLASMIC.
FT   TRANSMEM     28     47
FT   TOPO_DOM     48    428       NON CYTOPLASMIC.
//
```

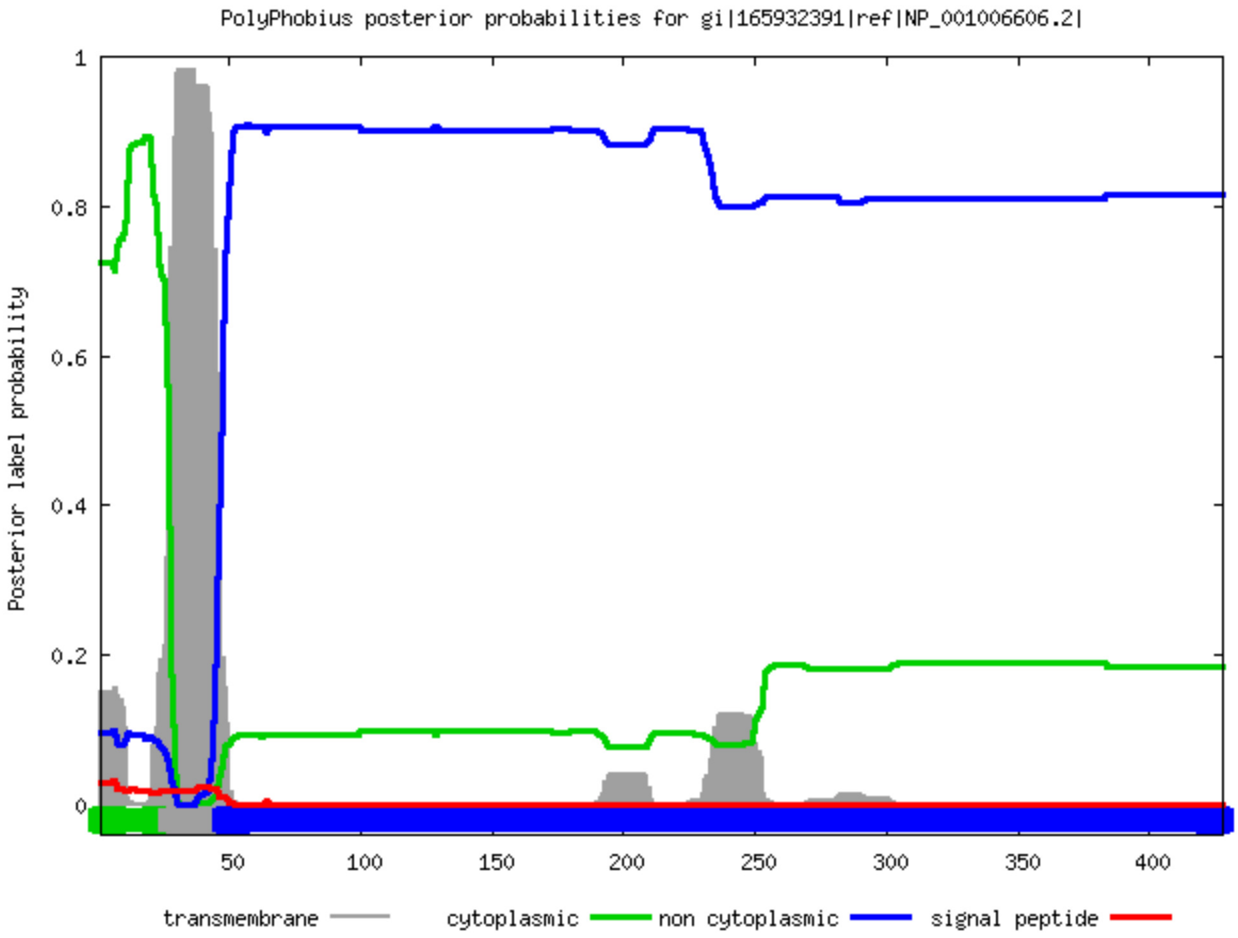

The prediction is based on an alignment. The probability data used in the plot is found here, and the gnuplot script is here.

Prediction of gi|27734895|ref|NP_775823.1|

```
ID   gi|27734895|ref|NP_775823.1|
FT   SIGNAL        1     37
FT   REGION        1     17       N-REGION.
FT   REGION       18     29       H-REGION.
FT   REGION       30     37       C-REGION.
FT   TOPO_DOM     38    430       NON CYTOPLASMIC.
//
```

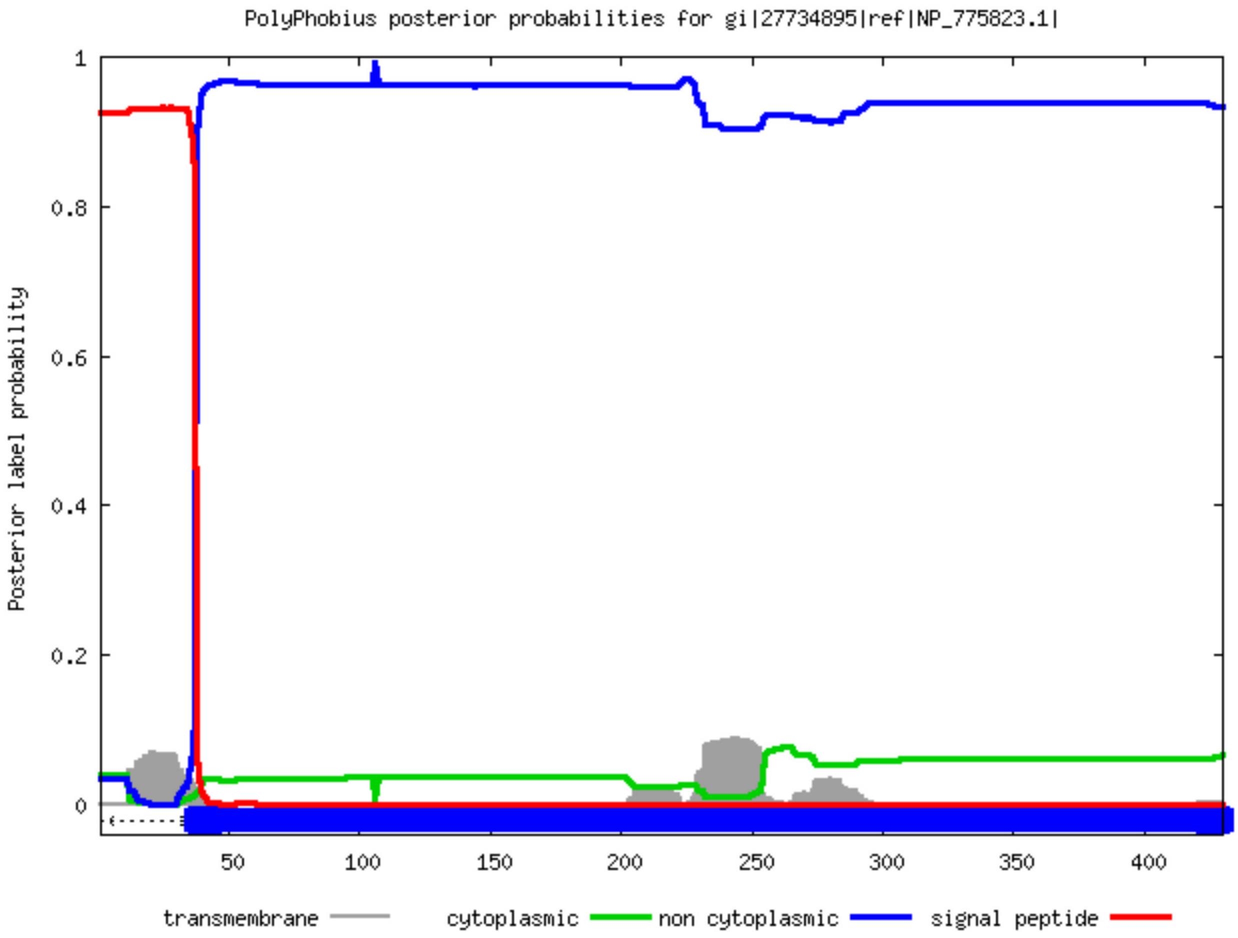

The prediction is based on an alignment. The probability data used in the plot is found here, and the gnuplot script is here.

Prediction of gi|193804854|ref|NP_789789.2|

```
ID   gi|193804854|ref|NP_789789.2|
FT   SIGNAL        1     34
FT   REGION        1     14       N-REGION.
FT   REGION       15     28       H-REGION.
FT   REGION       29     34       C-REGION.
FT   TOPO_DOM     35    433       NON CYTOPLASMIC.
//
```

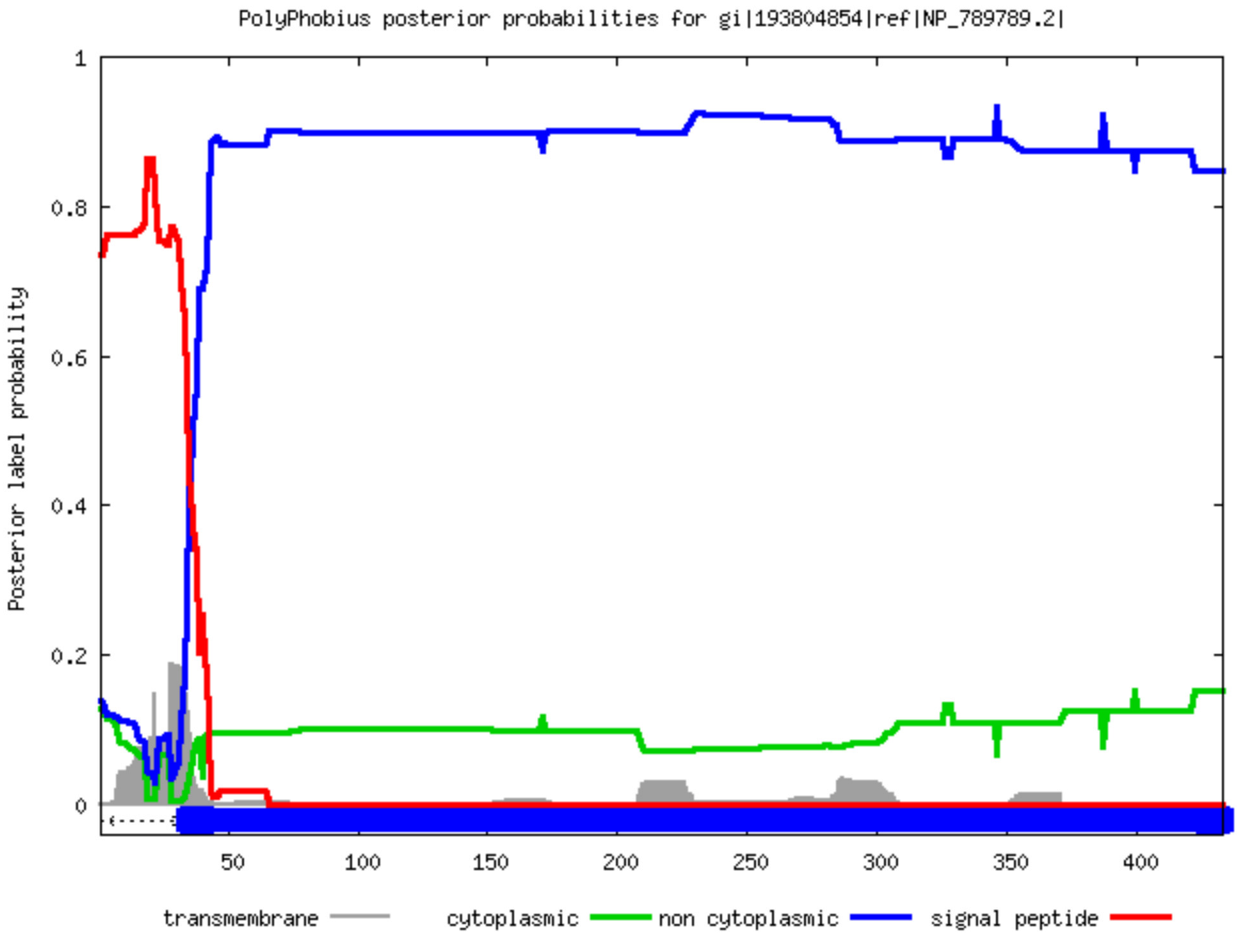

The prediction is based on an alignment. The probability data used in the plot is found here, and the gnuplot script is here.

Prediction of gi|224922839|ref|NP_689634.2|

```
ID   gi|224922839|ref|NP_689634.2|
FT   TOPO_DOM      1     30       CYTOPLASMIC.
FT   TRANSMEM     31     50
FT   TOPO_DOM     51    431       NON CYTOPLASMIC.
//
```

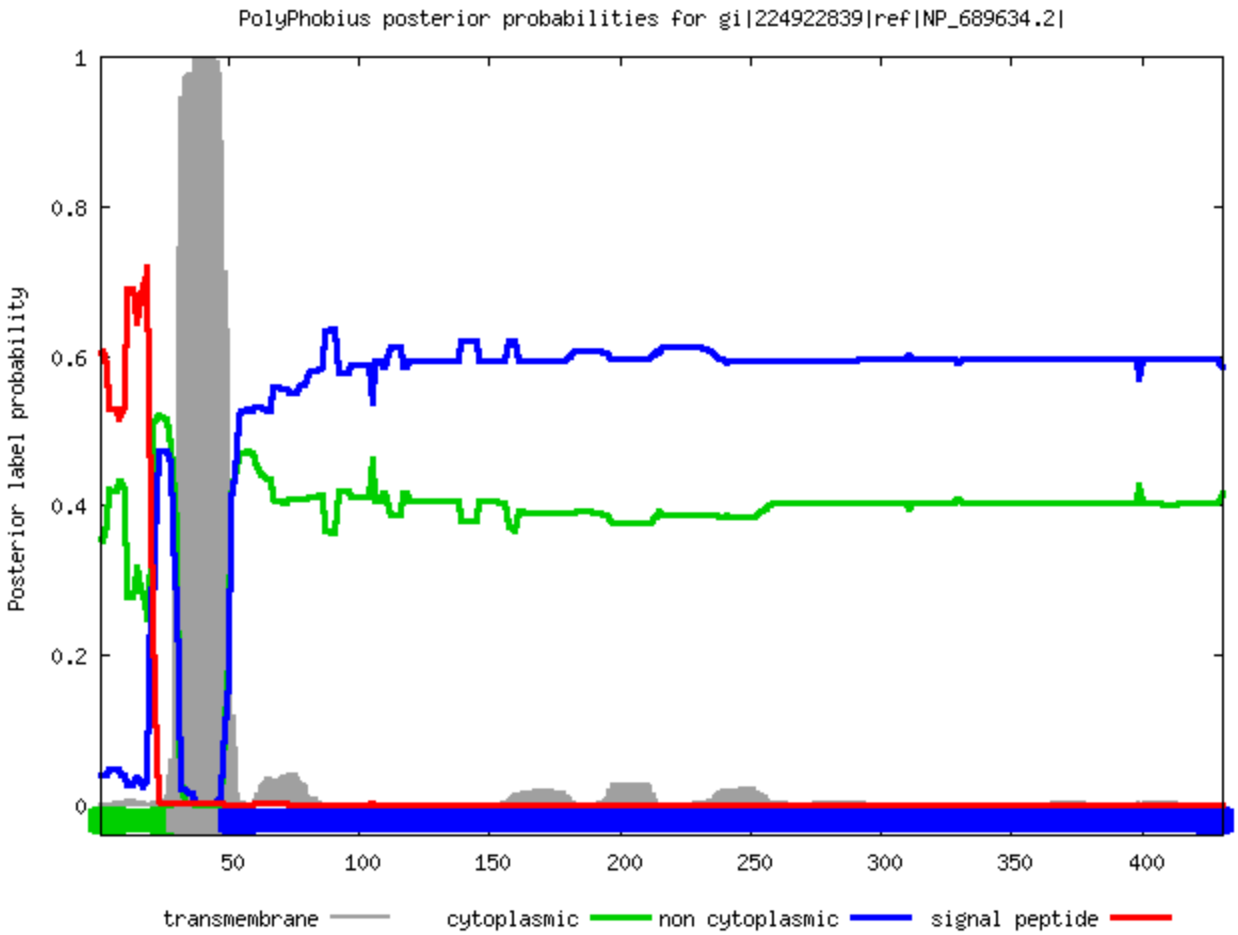

The prediction is based on an alignment. The probability data used in the plot is found here, and the gnuplot script is here.

Prediction of gi|195546915|ref|NP_001037834.2|

```
ID    gi|195546915|ref|NP_001037834.2|
FT    TOPO_DOM       1      23       CYTOPLASMIC.
FT    TRANSMEM      24      44
FT    TOPO_DOM      45     419       NON CYTOPLASMIC.
//
```

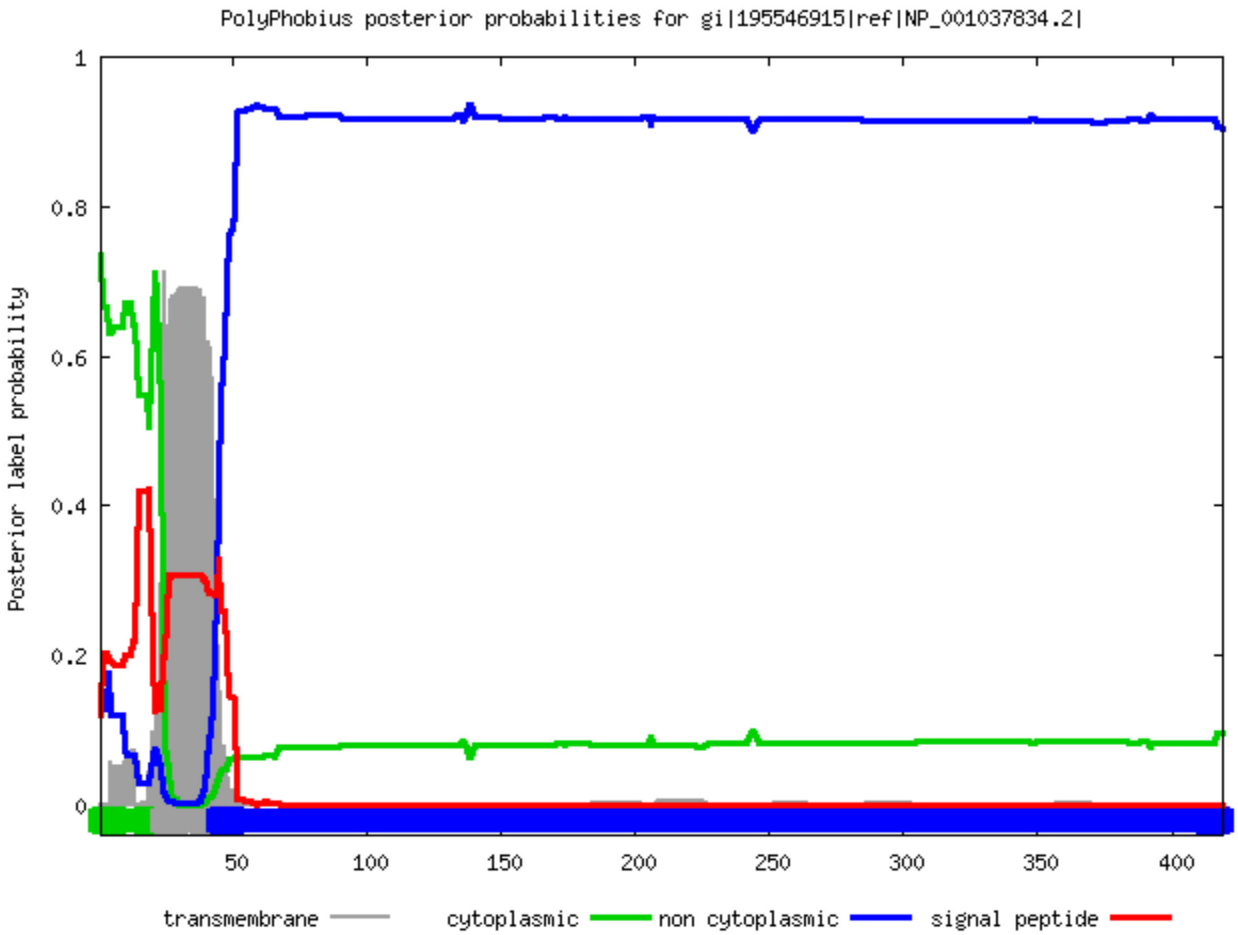

The prediction is based on an alignment. The probability data used in the plot is found here, and the gnuplot script is here.
